# Supplementary material for: Operando formation of highly efficient electrocatalysts induced by heteroatom leaching
Source: Nat Commun. 2024 Jan 4;15:242. doi: 10.1038/s41467-023-44480-9 (PMC10764338; doi:10.1038/s41467-023-44480-9)
Supplement: Supplementary file 1 — Supplementary Information [file 41467_2023_44480_MOESM1_ESM.pdf]

Supplementary Information

Operando formation of highly efficient electrocatalysts induced by heteroatom leaching

Cong Liu[1,2], Bingbao Mei[3], Zhaoping Shi[1,2], Zheng Jiang[2,3], Junjie Ge[1], Wei Xing[1,2], Ping Song[1,], and Weilin Xu[1,2]**

[1] State Key Laboratory of Electroanalytical Chemistry, & Jilin Province Key Laboratory of Low Carbon Chemical Power, Changchun Institute of Applied Chemistry, Chinese Academy of Sciences, Changchun 130022, China.

[2] School of Applied Chemistry and Engineering, University of Science and Technology of China, Hefei 230026, China.

[3] Shanghai Synchrotron Radiation Facility, Shanghai Institute of Applied Physics, Chinese Academy of Sciences, Shanghai 201204, China.

*Corresponding author. Email: songping@ciac.ac.cn; weilinxu@ciac.ac.cn

1. Supplementary Figures

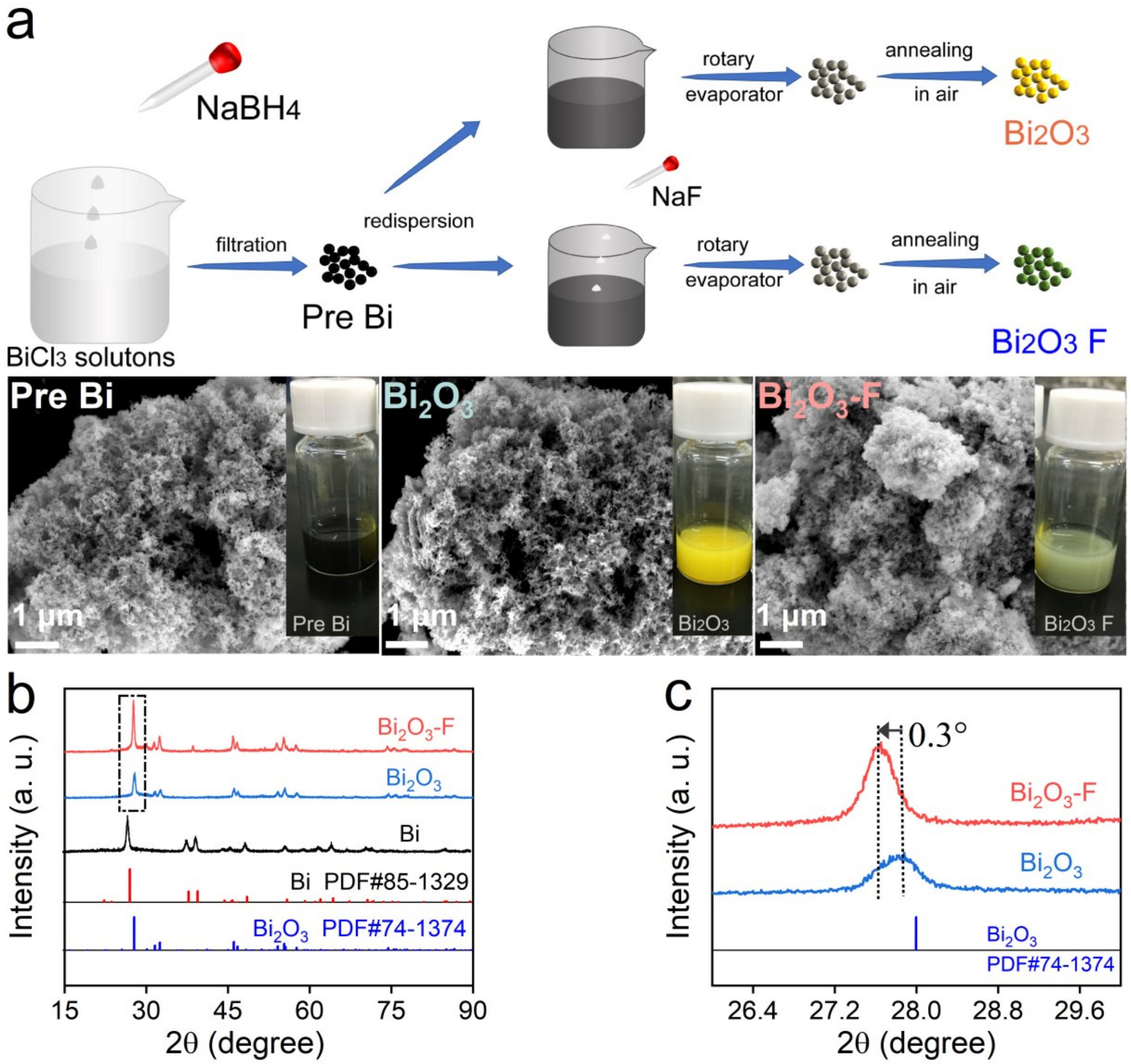

Supplementary Fig. 1. **Preparation process and X-ray Diffraction (XRD) results of catalyst.** (a) Schematic of the preparation of Pre Bi, Bi_2O_3, and Bi_2O_3-F and corresponding SEM images. The magnification is 1 μm. (b) XRD pattern comparison of Pre Bi, Bi_2O_3, and Bi_2O_3-F. Black, blue, and red line corresponds to Pre Bi, Bi_2O_3, and Bi_2O_3-F. (c) Enlarged comparison in the 25°-30° diffraction range of Bi_2O_3 and Bi_2O_3-F.

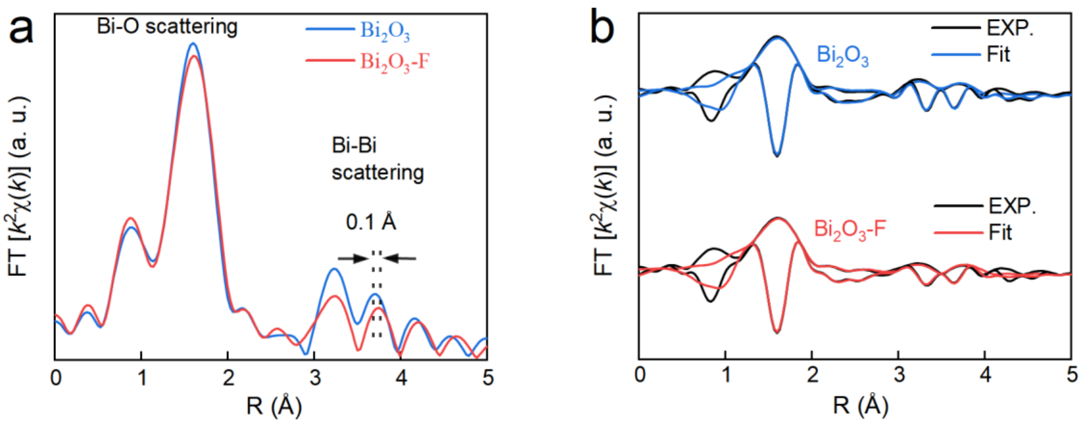

Supplementary Fig. 2. **X-ray Absorption Fine Structure (XAFS) analysis.** Extended x-ray absorption fine structure (EXAFS) spectra in R space (a) and corresponding fitting results (b), blue and red line corresponds to Bi_2O_3 and Bi_2O_3-F, respectively (a. u. = arbitrary units). The distance of Bi-Bi path of Bi_2O_3-F are larger than that of Bi_2O_3 indicate that the introduction of Fluorine increases the relaxation between Bi atomic layers and causes lattice expansion[1, 2].

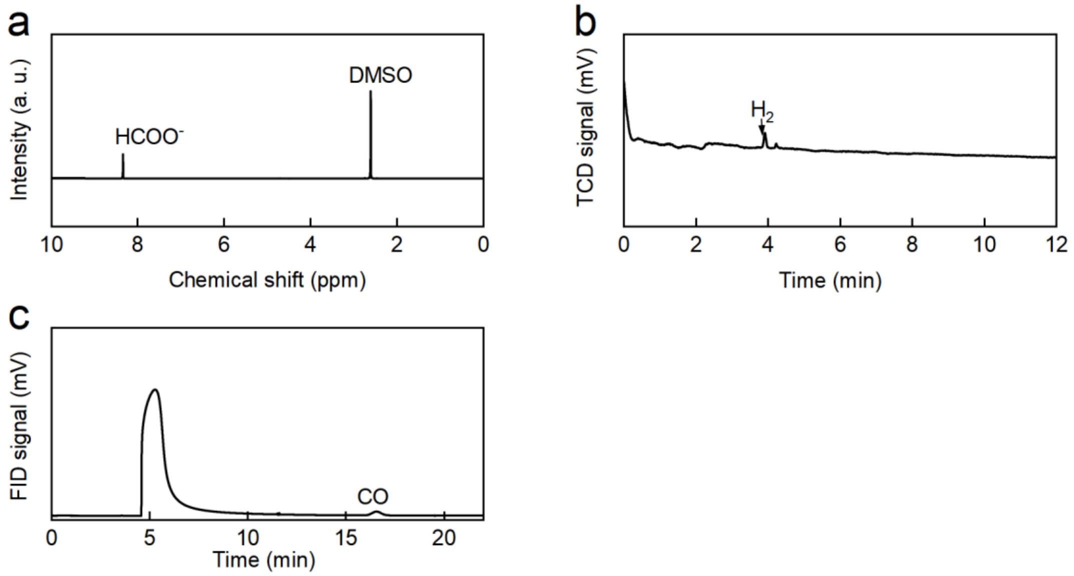

Supplementary Fig. 3. **An example of raw GC and NMR data for Bi_2O_3-F catalysts.** (a) NMR was used to determine the formate production. (b) and (c), The volume concentrations of H_2 and CO were detected by thermal conductivity detectors (TCD) and Flame ionization detectors (FID) respectively. These data were obtained from electrolysis experiments conducted at -1.27 V (RHE).

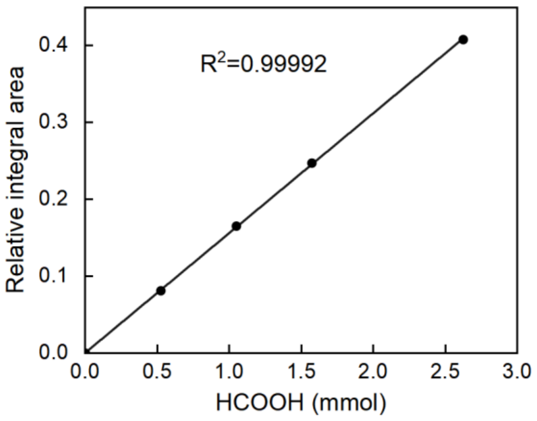

Supplementary Fig. 4. **$HCOO^-$ calibration curve.** Relative integral area based on the relative ratio between $HCOO^-$ and DMSO (internal standard).

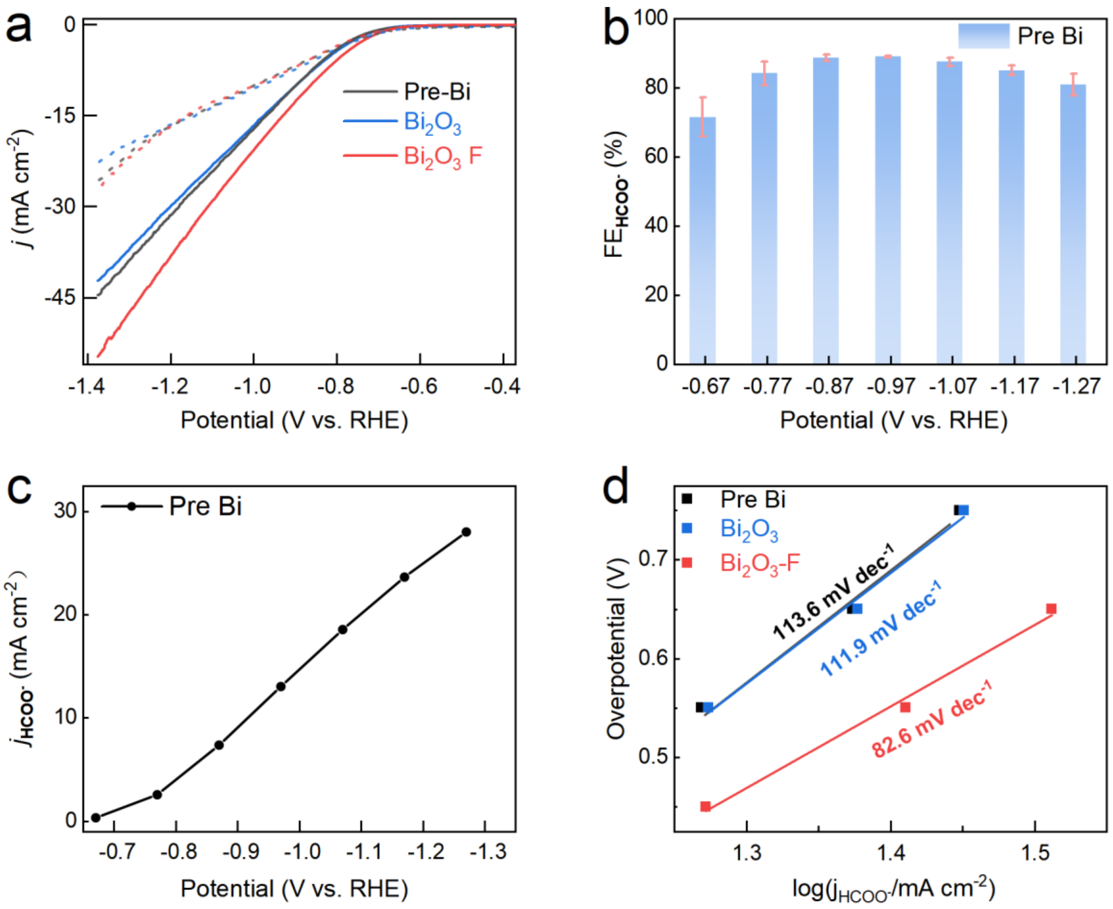

Supplementary Fig. 5. **The CO_2RR performance of Pre Bi.** (a) Comparison of LSV with pH corrections for CO_2 and Ar saturated electrolytes. Colours in black, blue, and red represent Pre Bi, Bi_2O_3, and Bi_2O_3-F, respectively. (b) FE_{HCOO-} and (c) j_{HCOO-} of Pre Bi at different applied potentials ranging from -0.67 V to -1.27 V (RHE). Error bars correspond to the standard deviation of three independent measurements. (d) Comparison of Tafel plots of formate production.

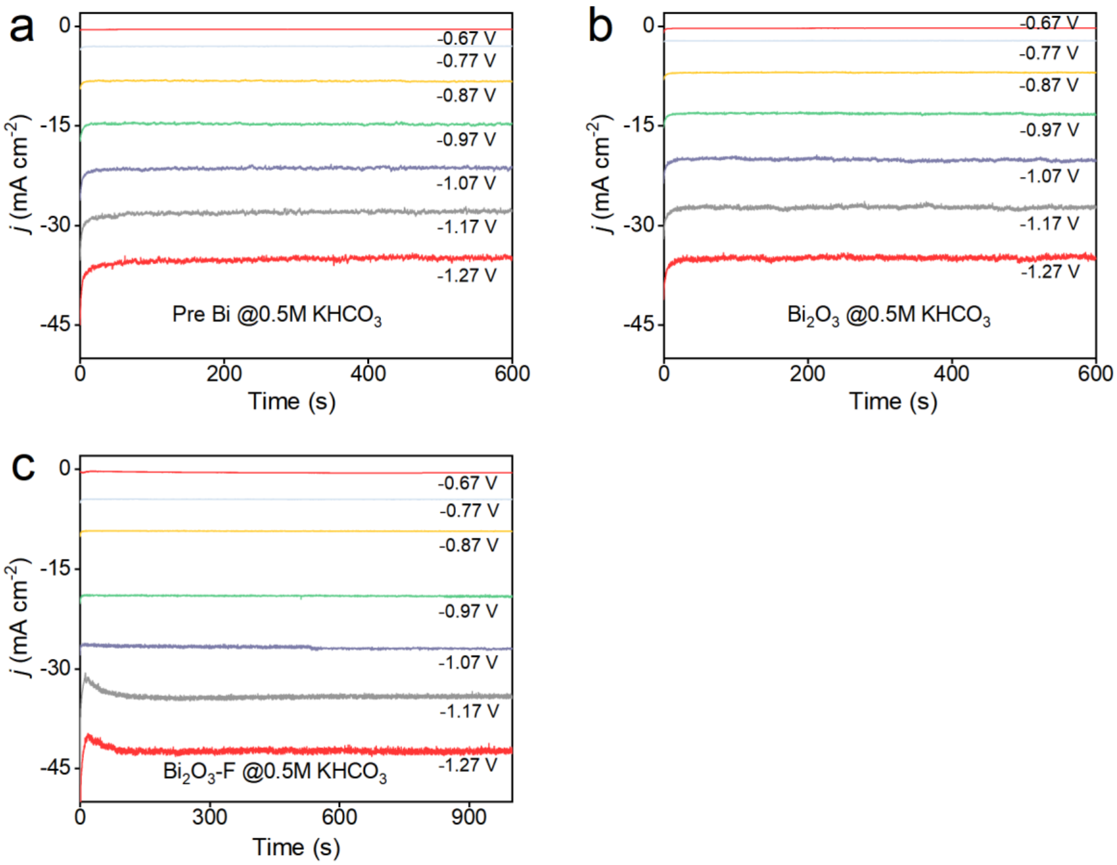

Supplementary Fig. 6. **Chronoamperometric responses at different potentials**. (a) Pre Bi, (b) Bi_2O_3, and (c) Bi_2O_3-F. Electrolyte: CO_2-saturated 0.5 M $KHCO_3$.

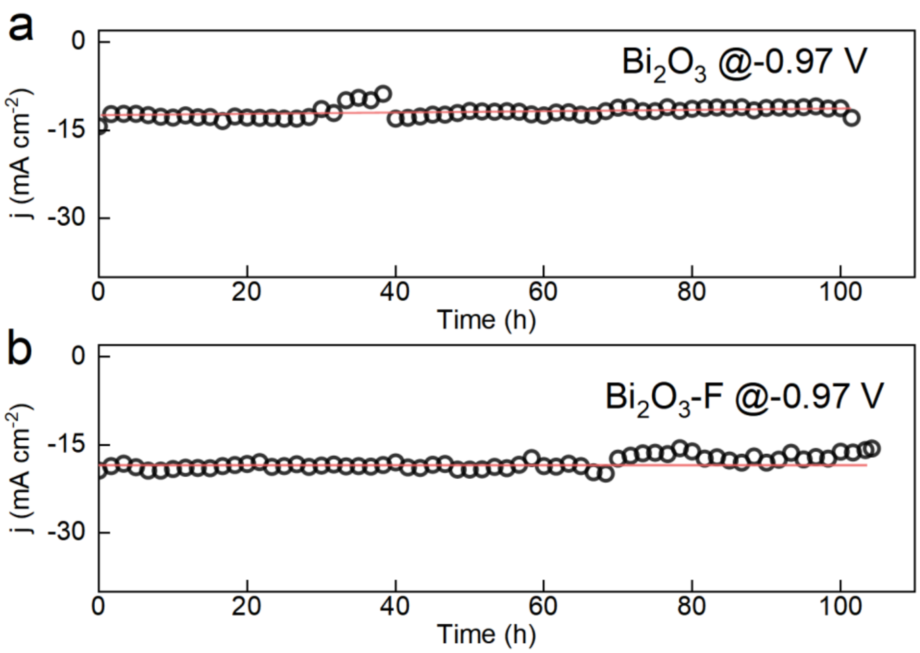

Supplementary Fig. 7. **Stability test results.** 100 h chronoamperometry curves of (a) Bi_2O_3 and (b) Bi_2O_3-F.

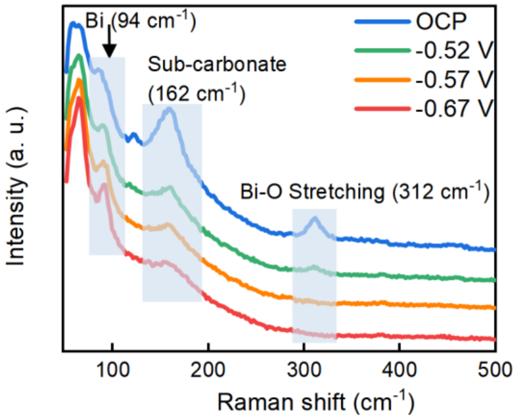

Supplementary Fig. 8. **Operando Raman spectra of Bi_2O_3.** Potential range from -0.52 to -0.67 V vs. RHE (CO_2 saturated 0.5 M $KHCO_3$) (a. u. = arbitrary units). Typical peaks around 162 cm^{-1} represent the embedment of CO_2 into the oxidic Bi_2O_3 matrix[3, 4] (sub-carbonate). The peak of sub-carbonates cannot be detected after the cathodic potential reaches -0.77 V, which is the onset potential for the detection of CO_2RR products.

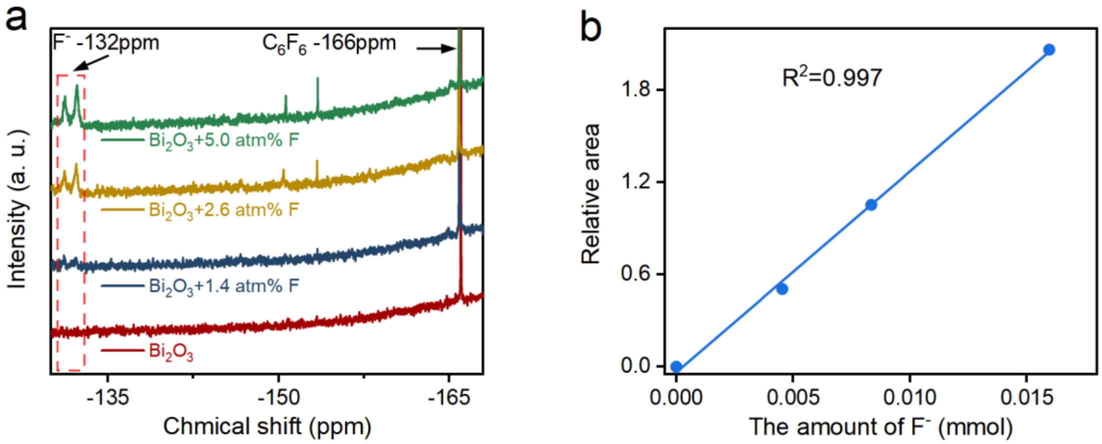

Supplementary Fig. 9. **The calibration curve of fluorine content.** (a) ^{19}F-NMR spectra of fluorine standard sample (NaF as fluorine source, hexafluorobenzene as internal standard). (b) The corresponding calibration curve obtained from (a).

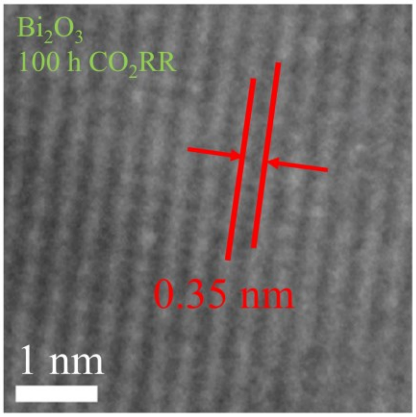

Supplementary Fig. 10. **Catalyst structure after catalytic reaction.** HRTEM images of Bi_2O_3 after 100 h CO_2RR (-0.97 V, 0.5 M $KHCO_3$). The lattice spacing analysis was attached.

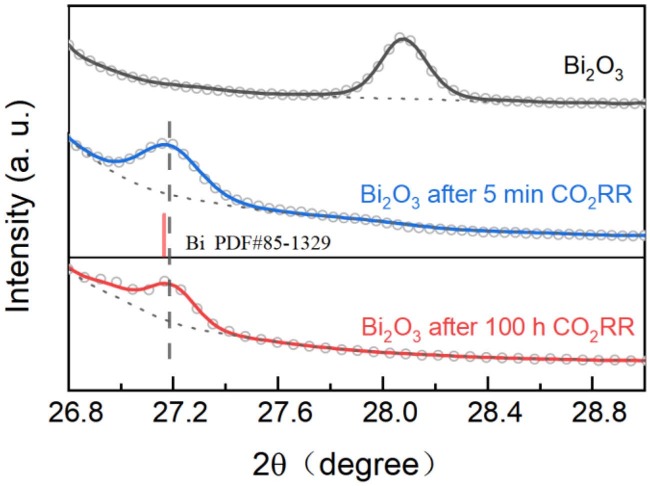

Supplementary Fig. 11. **Catalyst crystal structure after catalytic reaction.** Comparison of XRD refinement results of Bi_2O_3 and the catalyst after 5 min and 100 h CO_2RR in the region of 26.8-29 degree.

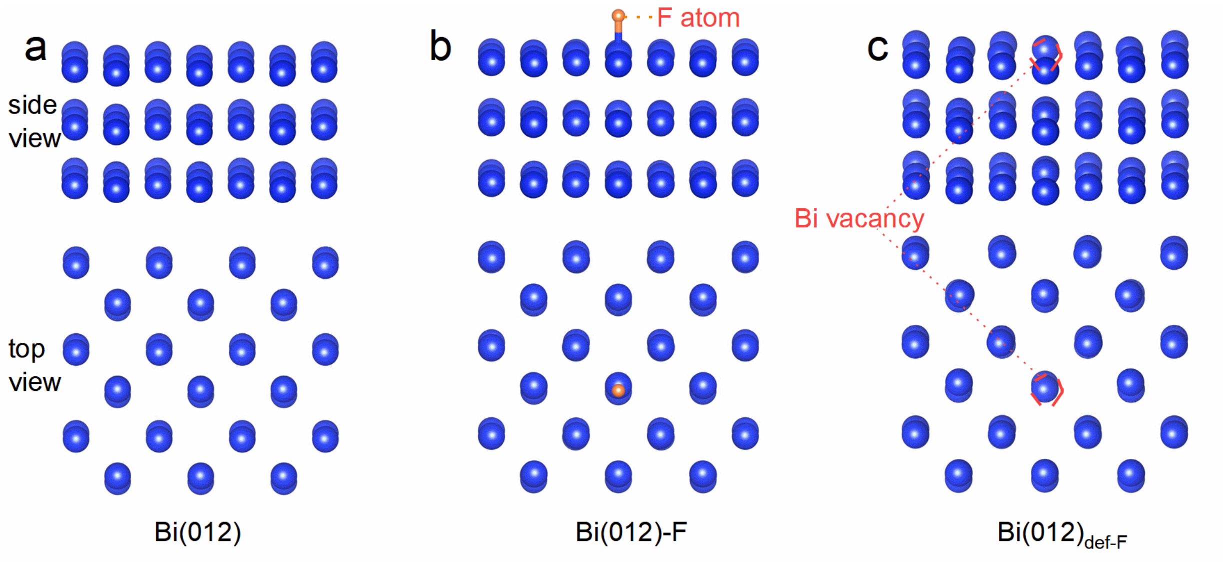

Supplementary Fig. 12. **Optimized geometric structures of atomic model.** (a) Bi(012) surface, (b) Bi(012)-F surface, and (c) Bi(012)$_{def\text{-}F}$ surface.

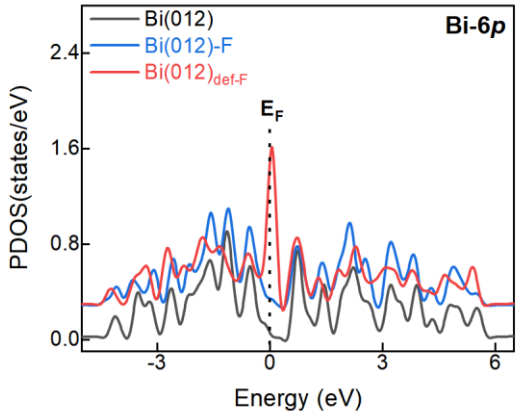

Supplementary Fig. 13. **Electronic structures of atomic model.** PDOSs of Bi-6*p* bands in the optimized geometric structures. Colours in black, blue, and red represent Bi(012) surface, Bi(012)-F surface, and Bi(012)$_{def\text{-}F}$ surface respectively.

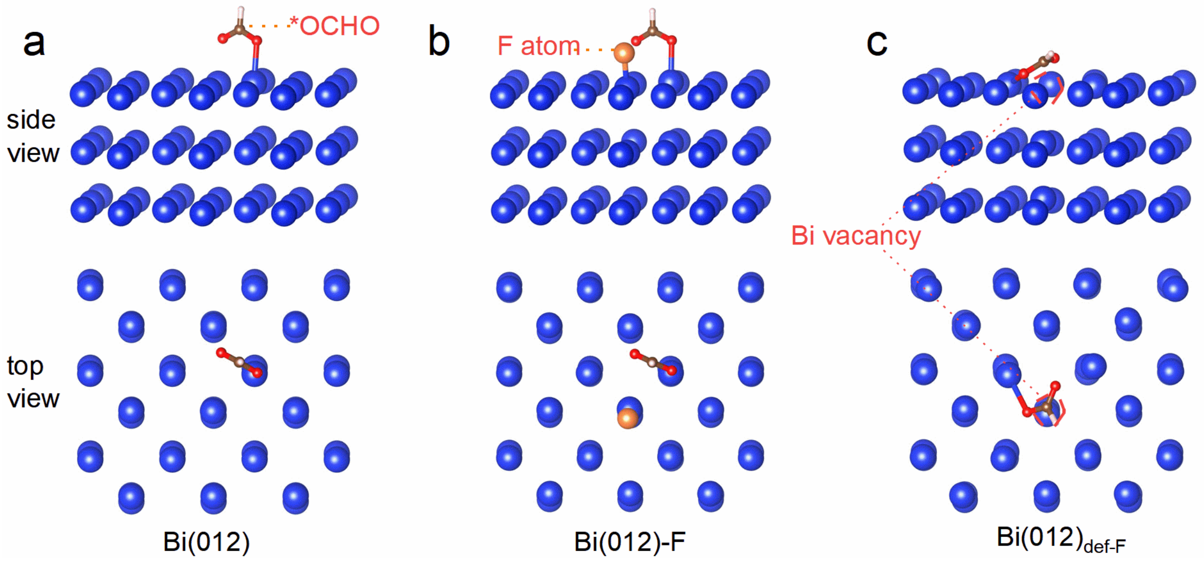

Supplementary Fig. 14. **Optimized adsorption structures of *OCHO.** (a) Bi(012) surface, (b) Bi(012)-F surface, and (c) Bi(012)$_{def\text{-}F}$ surface.

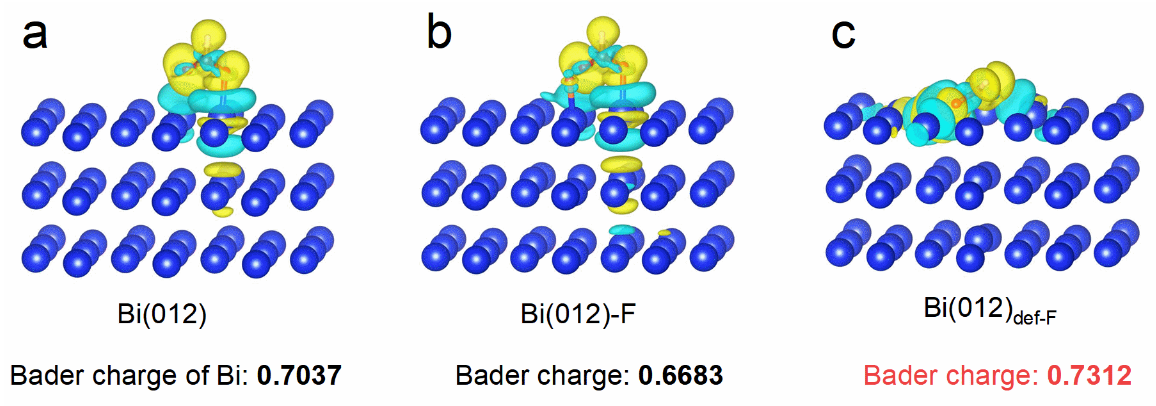

Supplementary Fig. 15. **The charge density difference and the electron transfer (bader charge) in each intermediate-adsorbed Bi-based catalyst.** (a) Bi(012) surface, (b) Bi(012)-F surface, and (c) Bi(012)$_{def\text{-}F}$ surface. The yellow color represents charge accumulation and blue represents charge dissipation in space. The introduction of F reduces the electron transfer of intermediate-adsorbed Bi(012)-F surface. The formation of defects increases the electron transfer of intermediate-adsorbed Bi(012)$_{def\text{-}F}$.

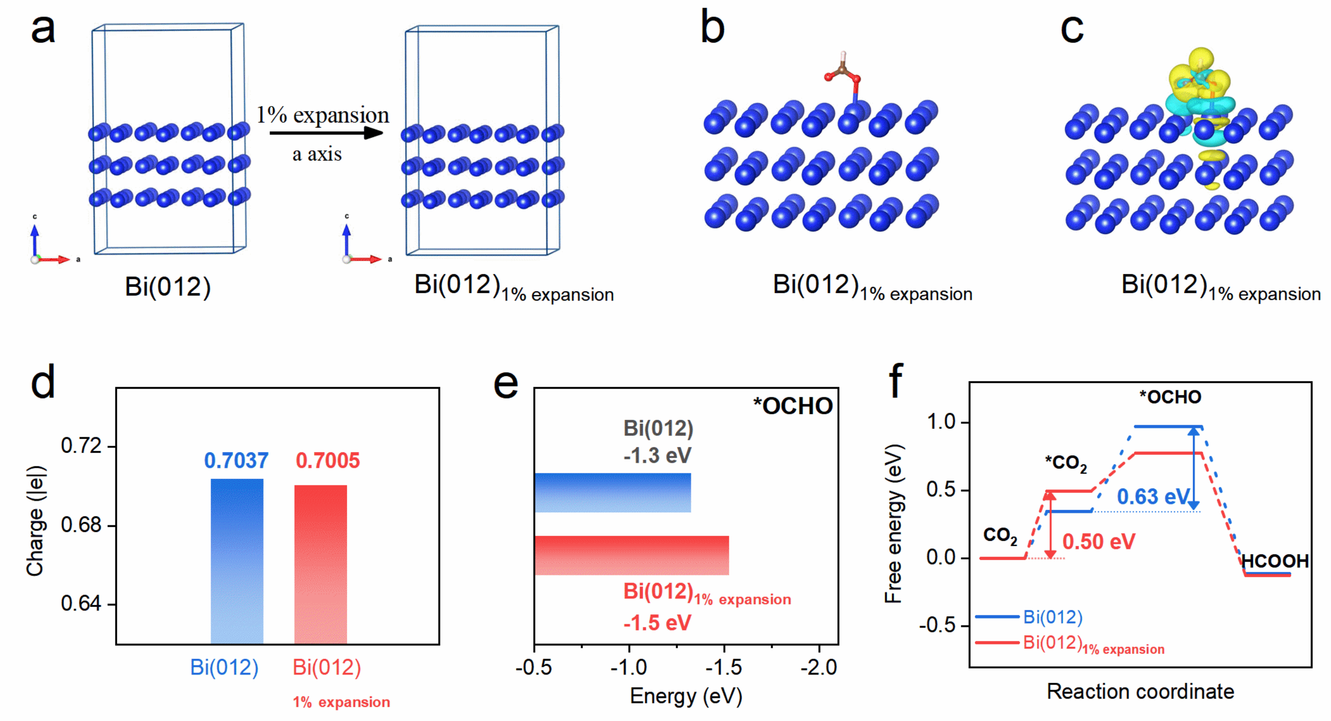

Supplementary Fig. 16. **Theoretical calculations of lattice expansion effects.** (a) The lattice of Bi(012) was expanded by 1% along the a-axis to investigate the effect of lattice strain on the CO_2RR (denoted as $Bi(012)_{1\%\text{-expansion}}$). (b) Optimized adsorption structures of *OCHO for $Bi(012)_{1\%\text{-expansion}}$ surface. (c) The charge density difference in intermediate-adsorbed $Bi(012)_{1\%\text{-expansion}}$ surface. (d) Bader charge of active sites and (e) binding energy of *OCHO in the different catalytic surface (Bi(012) and $Bi(012)_{1\%\text{-expansion}}$). (f) Free energy profiles for the formation of *OCHO intermediate on the catalytic surface. Colours in blue and red represent Bi(012) surface, $Bi(012)_{1\%\text{-expansion}}$ surface respectively.

Supplementary note 1: Based on the calculation results of charge density difference and bader charge (Supplementary Fig. 16), there is no significant change in the electron transfer of intermediate-adsorbed $Bi(012)_{1\%\ expansion}$ surface as compared to the Bi(012) surface (from the catalytic surface to the *OCHO intermediate). The results of intermediate adsorption energy showed that the *OCHO adsorption of $Bi(012)_{1\%\ expansion}$ was enhanced compared to the Bi(012) but still weaker than that of $Bi_{def\text{-}F}$ (Supplementary Fig. 16 and Figure 3c). The free energy profiles for the formation of *OCHO intermediate revealed that the rate-determining step of $Bi(012)_{1\%\ expansion}$ changes to the CO_2 activation (*+CO_2→*CO_2, energy barrier 0.5 eV) as compared to the Bi(012) (*CO_2 to *OCHO, energy barrier 0.63 eV). Nevertheless, the $Bi_{def\text{-}F}$ had the optimal intermediate step (*CO_2 to *OCHO, energy barrier 0.09 eV), and the CO_2 activation was the rate-determining step (energy barrier 0.33 eV), which owns the best catalytic activity among the four models. In general, the effect of lattice expansion on the electron transfer of intermediate-adsorbed $Bi(012)_{1\%\ expansion}$ surface is minimal. The change in lattice strain of $Bi(012)_{1\%\ expansion}$ causes the optimization of the intermediate step (*CO_2 to *OCHO), yet at the same time increases the energy barrier of the CO_2 activation process, which is a trade-off effect. More importantly, the CO_2RR activity of the $Bi_{def\text{-}F}$ was significantly altered by the electronic effects (enhanced electron transfer) and therefore the defects caused by the F leaching are considered as major factor for the enhanced activity observed in the experiment.

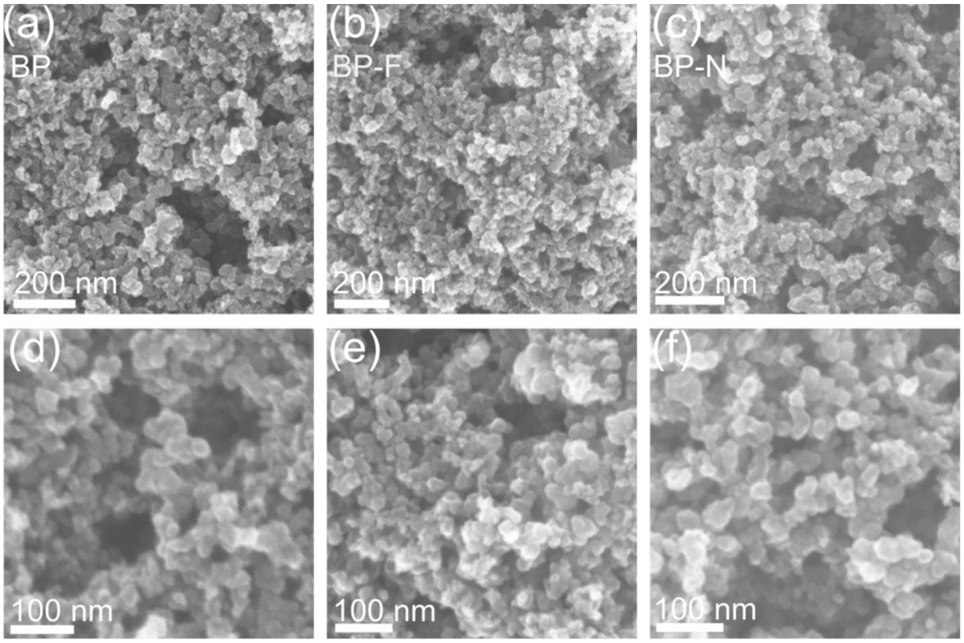

Supplementary Fig. 17. **SEM images of catalyst.** (a,d) BP, (b,e) BP-F, and (c,f) BP-N.

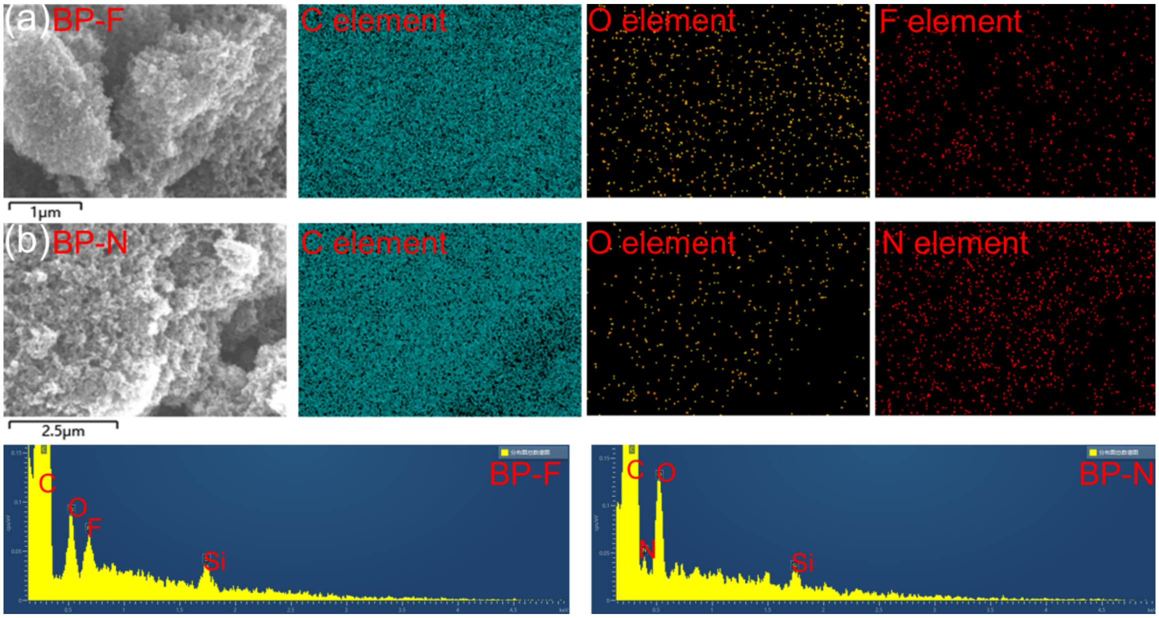

Supplementary Fig. 18. **EDS elemental mapping on SEM.** (a) BP-F and (b) BP-N.

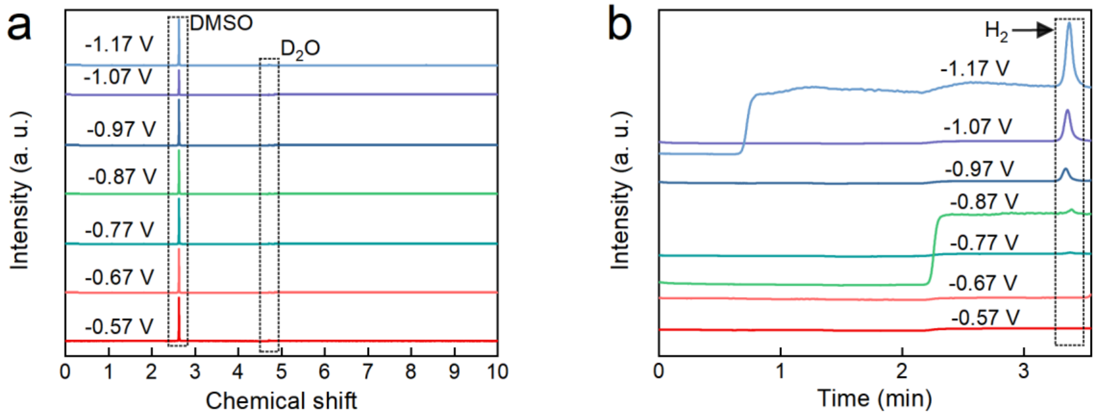

Supplementary Fig. 19. **CO_2RR performance of pure BP.** (a) ^{1}H-NMR results show no liquid product at different potentials (CO_2-saturated 0.5 M $KHCO_3$). (b) GC results (TCD) show no gas product apart from H_2 (a. u. = arbitrary units). The pure carbon black (BP) is inert to CO_2RR.

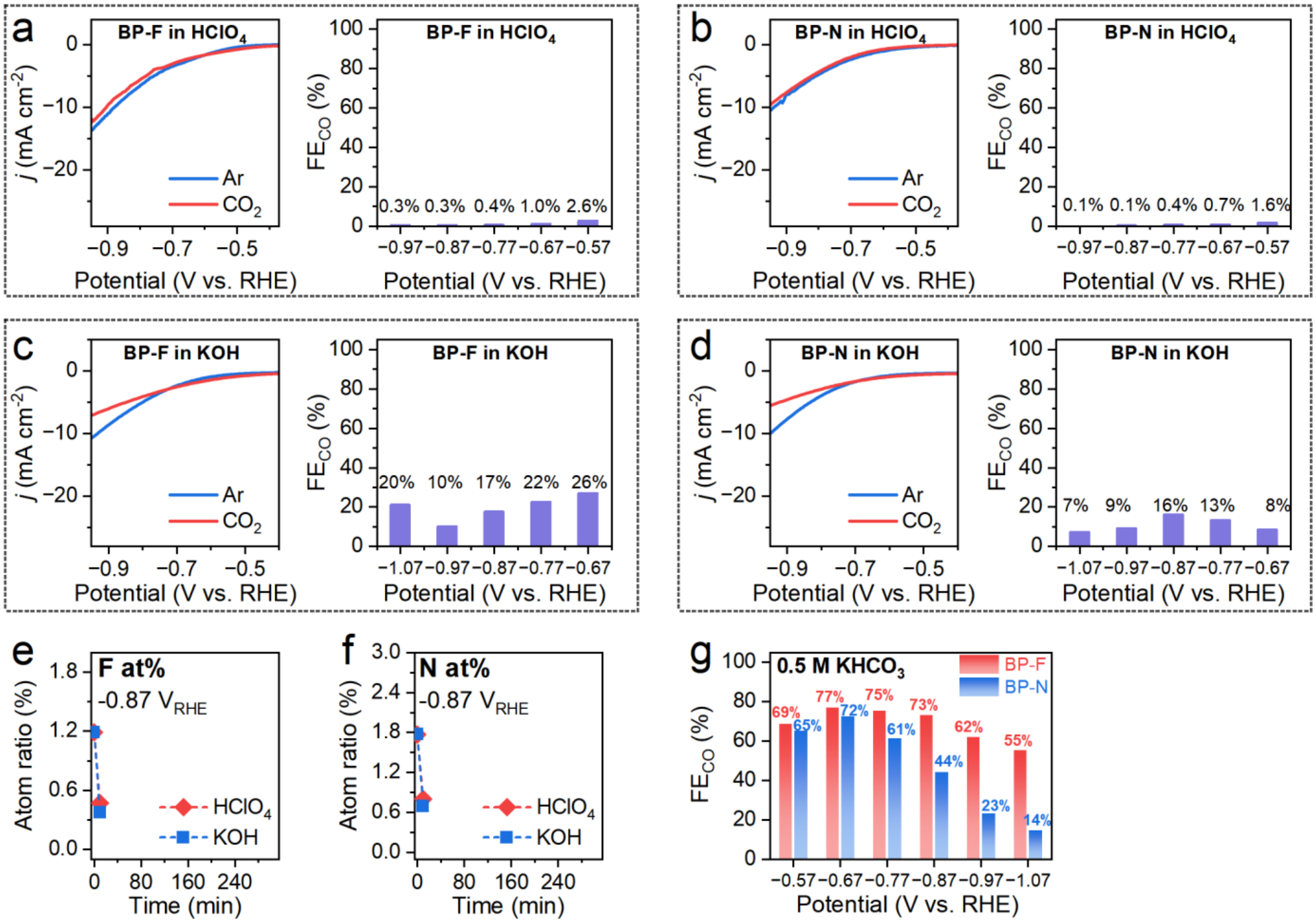

Supplementary Fig. 20. **The study of CO_2RR under overly basic or acidic conditions.** (a)-(d) LSV and corresponding FE_{CO} at different applied potentials of BP-F and BP-N in 0.1 M $HClO_4$ and 0.1 M KOH. Colours in red and blue represent CO_2 and Ar saturated electrolytes respectively. (e) F-dopant and (f) N-dopant content as a function of time of the potential (-0.87 V) applied in 0.1 M $HClO_4$ (red) and 0.1 M KOH (blue). (g) Comparison of FE_{CO} at different applied potentials ranging from -0.57 V to -1.07 V (RHE) in CO_2 saturated 0.5 M $KHCO_3$. Colours in red and blue represent BP-F and BP-N respectively. For the H-cell test, $KHCO_3$ is the optimal choice of electrolyte for the CO_2RR. The study of CO_2RR under overly basic (ex KOH) or acidic conditions (ex $HClO_4$) was not considered due to the poor CO_2RR performance. Moreover, supplementary Fig. 20e,f also show the fast leaching of N and F even in KOH and $HClO_4$, approximately the same as that in $KHCO_3$ solution (Fig. 4e,g), confirming that the electrode potential is the main reason for the leaching of these dopants.

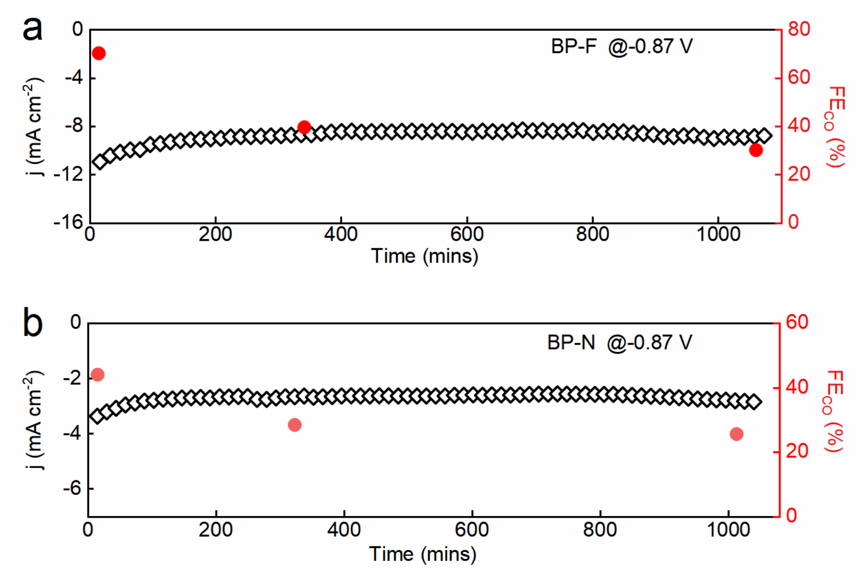

Supplementary Fig. 21. **The long-term stability and product selectivity of CO_2RR.** (a) BP-F and (b) BP-N (CO_2-saturated 0.5 M $KHCO_3$, -0.87 V RHE). In the 1000 minutes testing window, BP-F and BP-N maintain the catalytic activity for CO_2RR.

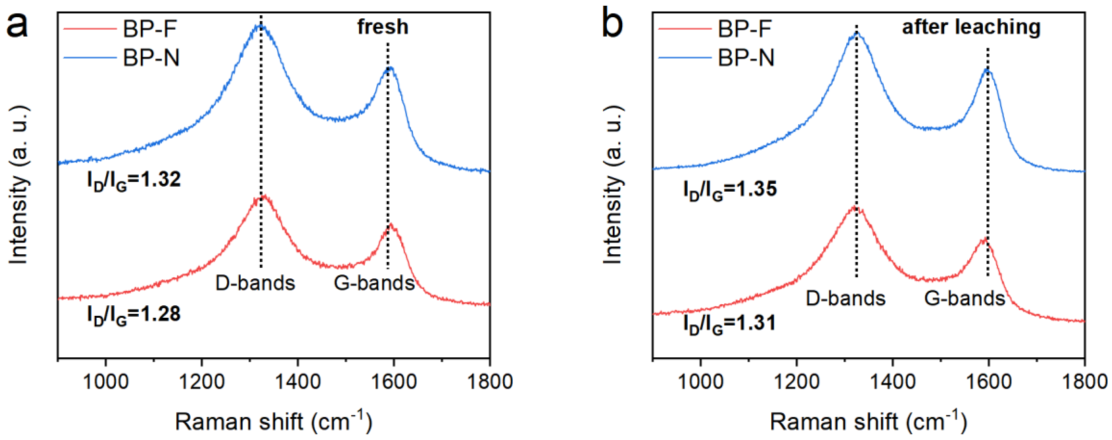

Supplementary Fig. 22. **Raman characterisation of the catalyst before and after dopant leaching.** (a) Raman spectra of fresh BP-F and BP-N. (b) Raman spectra of BP-F and BP-N after doapnt leaching (CO_2-saturated $KHCO_3$, -0.87 V, 60 min) (a. u. = arbitrary units). Colours in red and blue represent BP-F and BP-N respectively. After the dopant leaching (a low voltage is used), the defect degree of BP-F and BP-N is further increased from the present level.

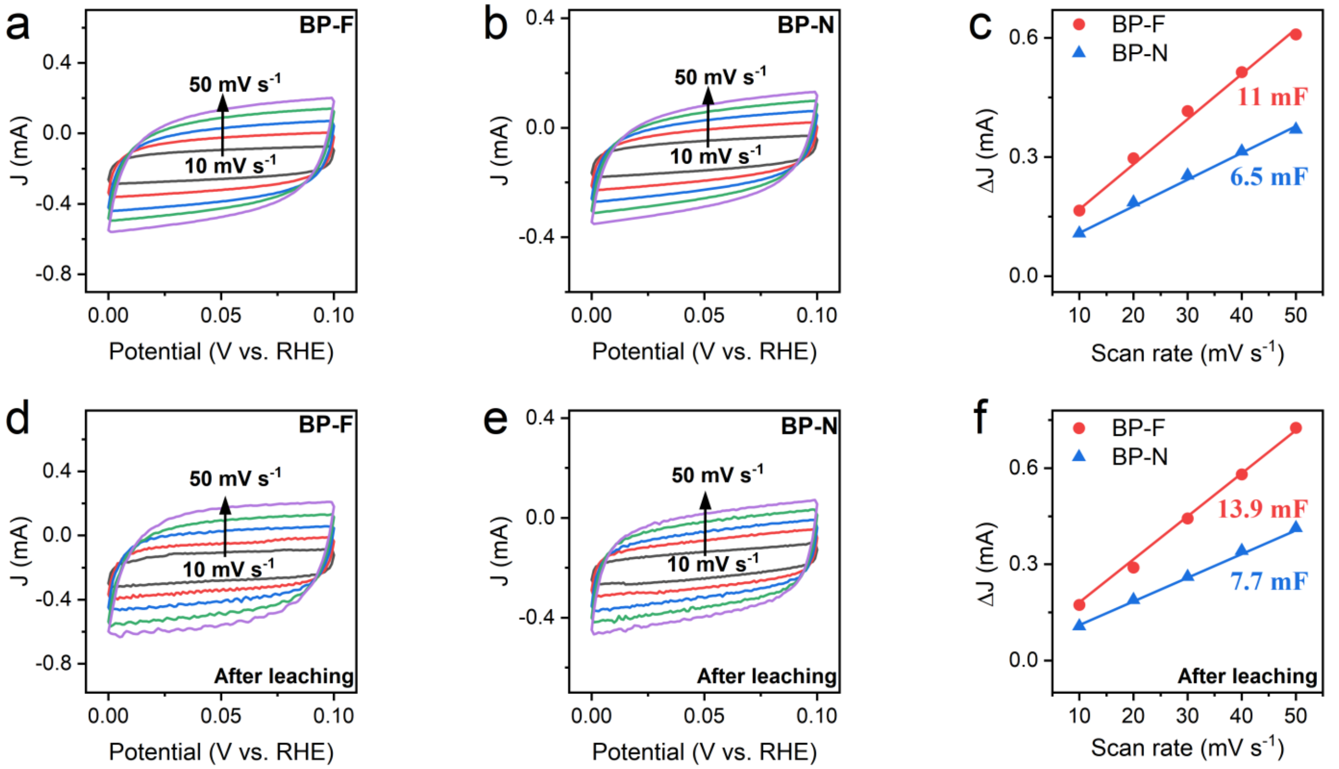

Supplementary Fig. 23. **Electrochemical double layer capacitance of the catalyst before and after dopant leaching.** (a)-(c) CV profiles of BP-F and BP-N in the non-Faradaic region of 0 – 0.1 V vs. RHE with the scan rate of 10, 20, 30, 40, 50 mV s^{-1} and corresponding electrochemical double layer capacitance (C_{dl}) plots obtained from the CV curves. (d)-(f) The C_{dl} test results after the dopant leaching (CO_2-saturated $KHCO_3$, -0.87 V, 60 min.). The test results of the double layer capacitance demonstrated the electrochemical active surface area (ECSA) of the BP-F to be 1.7 times higher than that of the BP-N, reaching 1.8 times higher after the dopant leaching. The ECSA as well as the different types of active sites together influence the activity and selectivity of CO_2RR[5-7].

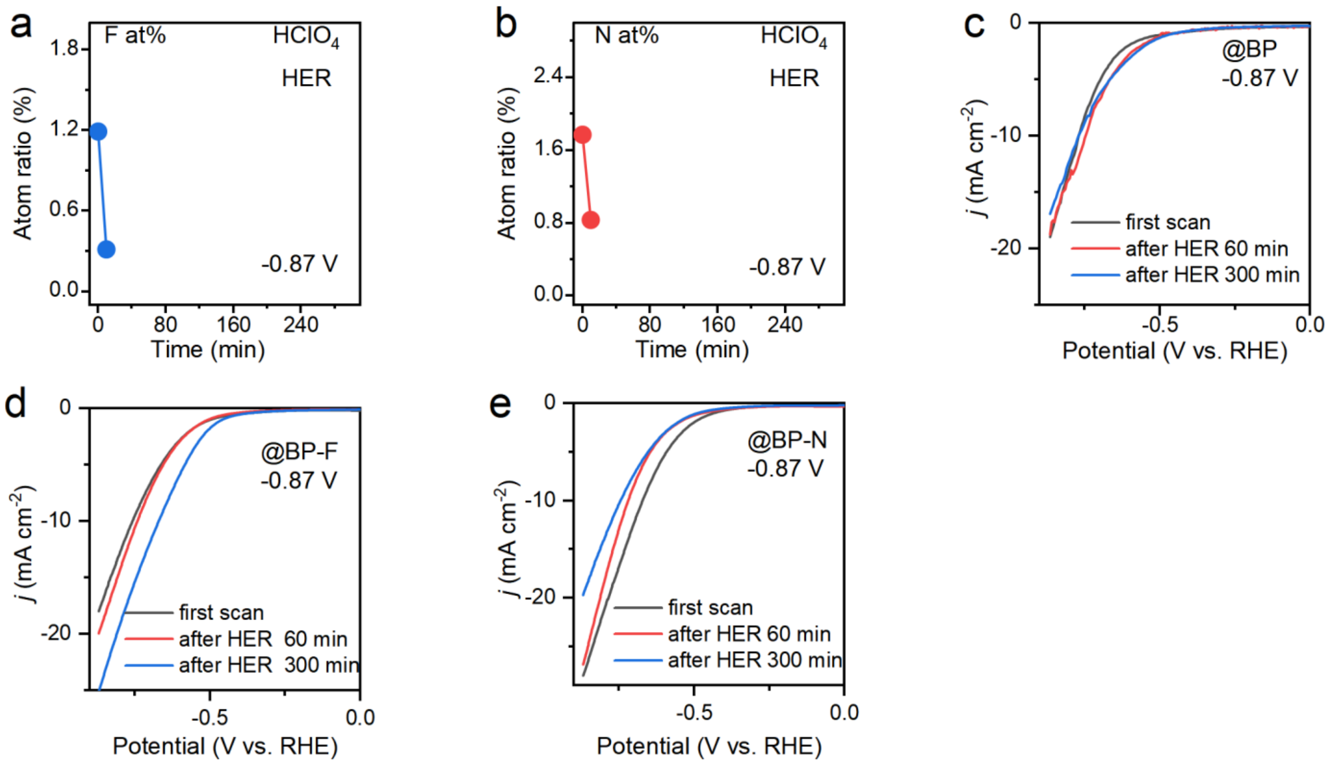

Supplementary Fig. 24. **Changes in catalyst activity before and after the dopant leaching.** (a) F-dopant content and (b) N-dopant content as a function of potential applied time under -0.87 V vs. RHE (in Ar-saturated 0.1 M $HClO_4$). The varying of HER activity of (c) BP, (d) BP-F, and (e) BP-N after a certain time chronoamperometry test in the leaching potential (-0.87 V_{RHE}).

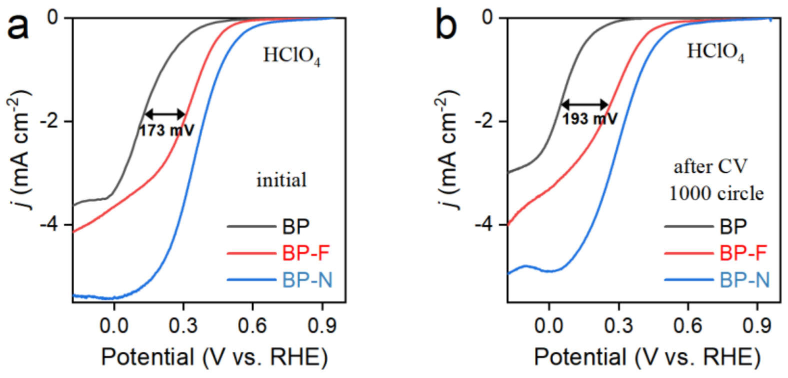

Supplementary Fig. 25. **The oxygen reduction reaction (ORR) performance of BP, BP-F, and BP-N.** (a) The initial catalytic performance of BP, BP-F, and BP-N. (b) The catalytic performance of BP, BP-F, and BP-N after cyclic voltammetry (CV) test (1000 circle). For stability test, the CV scan rate was 50 mV/s.

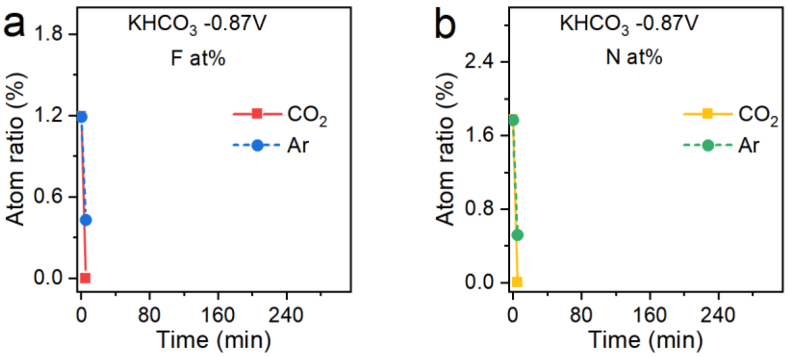

Supplementary Fig. 26. **Comparisons of leaching rates between the different reaction types (CO_2RR and HER).** The varying of (a) F and (b) N atom ratios in BP under -0.87 V vs. RHE (solid line and dash line were tested in CO_2-saturated and Ar-saturated 0.5 M $KHCO_3$, respectively).

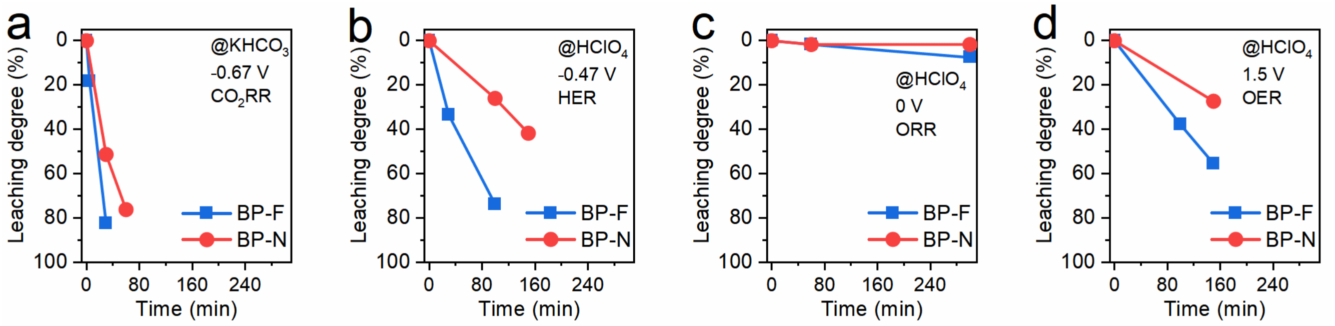

Supplementary Fig. 27. **Comparisons of leaching rates between the different types of dopants (F-dopants and N-dopants).** The experimental conditions were (a) CO_2RR, (b) HER, (c) ORR, and (d) OER, respectively. The CO_2RR was tested in CO_2-saturated 0.5 M $KHCO_3$ (-0.67 V vs. RHE), the HER was tested in Ar-saturated 0.1 M $HClO_4$ (-0.47 V vs. RHE), the ORR were tested in O_2-saturated 0.1 M $HClO_4$ (0 V vs. RHE), and the OER were tested in O_2-saturated 0.1 M $HClO_4$ (1.5 V vs. RHE). The slower leaching rate of N-dopant may be correlated with the type of C-N bond (Pyrrole nitrogen)[8, 9].

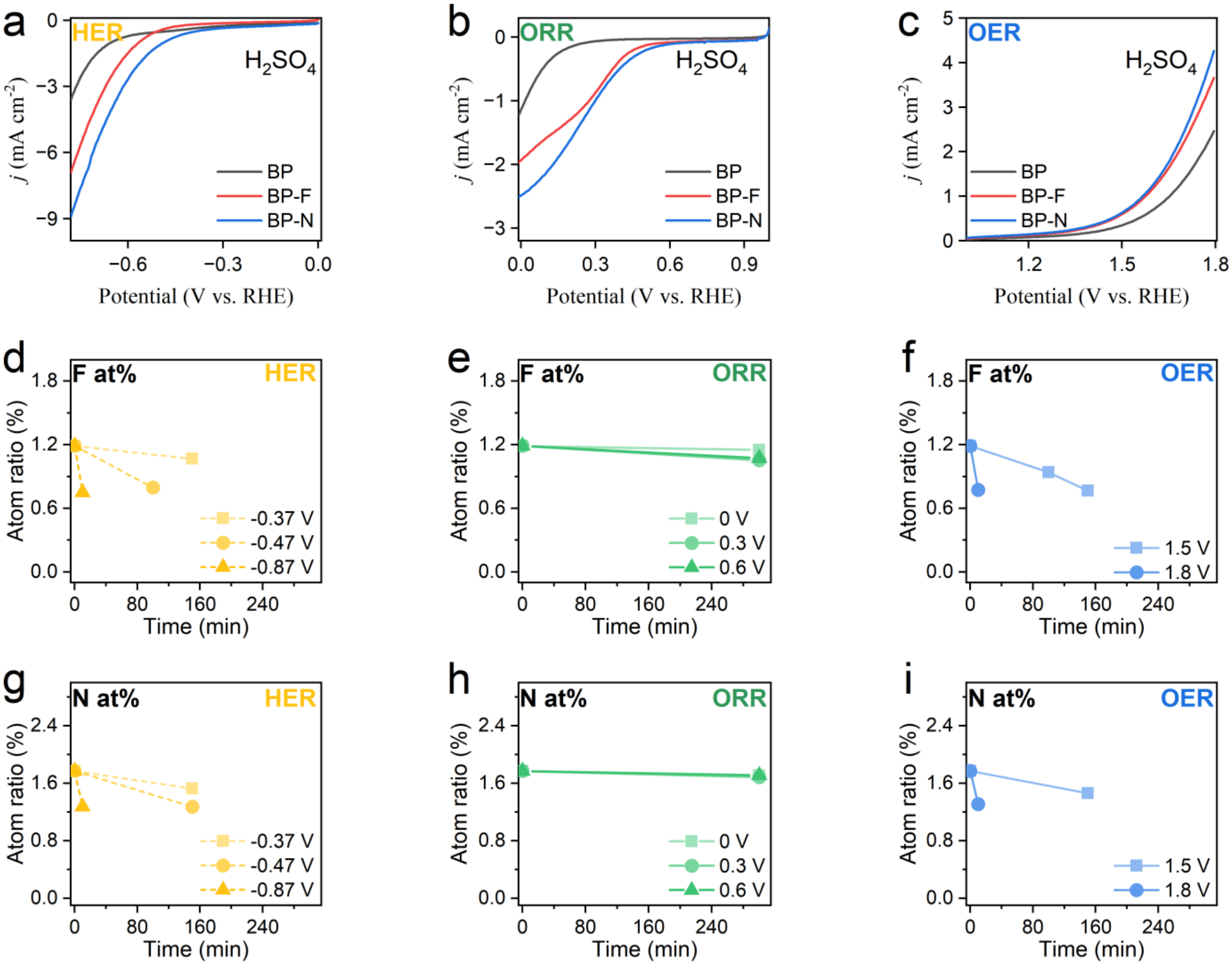

Supplementary Fig. 28. **Dopant leaching in sulphuric acid electrolytes.** (a-c) The linear sweep curves of the catalyst (rotation rate 1600 rpm, scan rate of 5 mV/s). (a) HER was tested in Ar-saturated 0.05 M H_2SO_4, (b) ORR and (c) OER were tested in O_2-saturated 0.05 M H_2SO_4. Colours in black, red, and blue represent BP, BP-F, and BP-N, respectively. (d-f) F-dopant and (g-i) N-dopant content as a function of potential applied time in the 0.05 M H_2SO_4, (d, g) HER, (e, h) ORR, and (f, i) OER. The similar leaching mechanism was found in the 0.05 M H_2SO_4. The dopant leaching occurs in both the HER and OER test intervals, while there is no significant leaching in the ORR test interval.

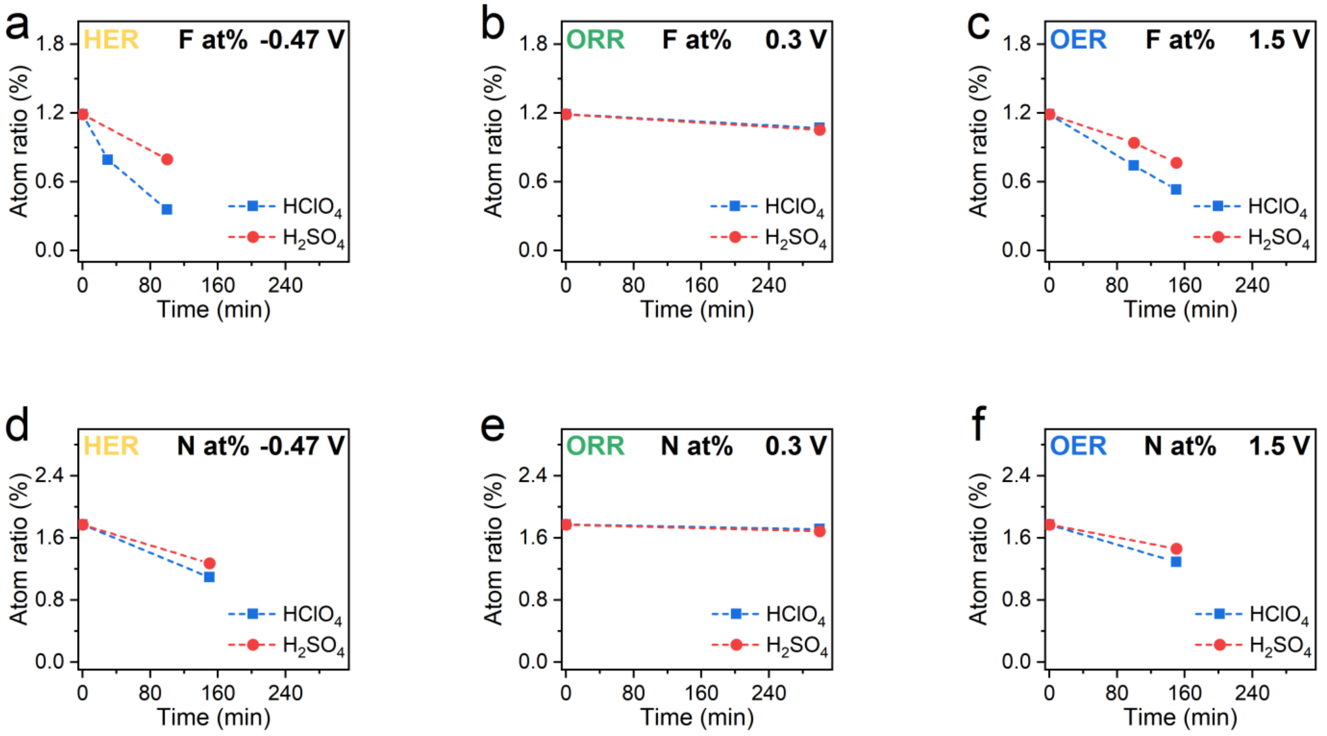

Supplementary Fig. 29. **Comparisons of leaching rates between the different electrolytes.** Comparisons of (a-c) F-dopant and (d-f) N-dopant content variations in the different electrolyte at same applied potential. In the HER and OER test intervals, the dopant leaching is slowed down in H_2SO_4 electrolytes.

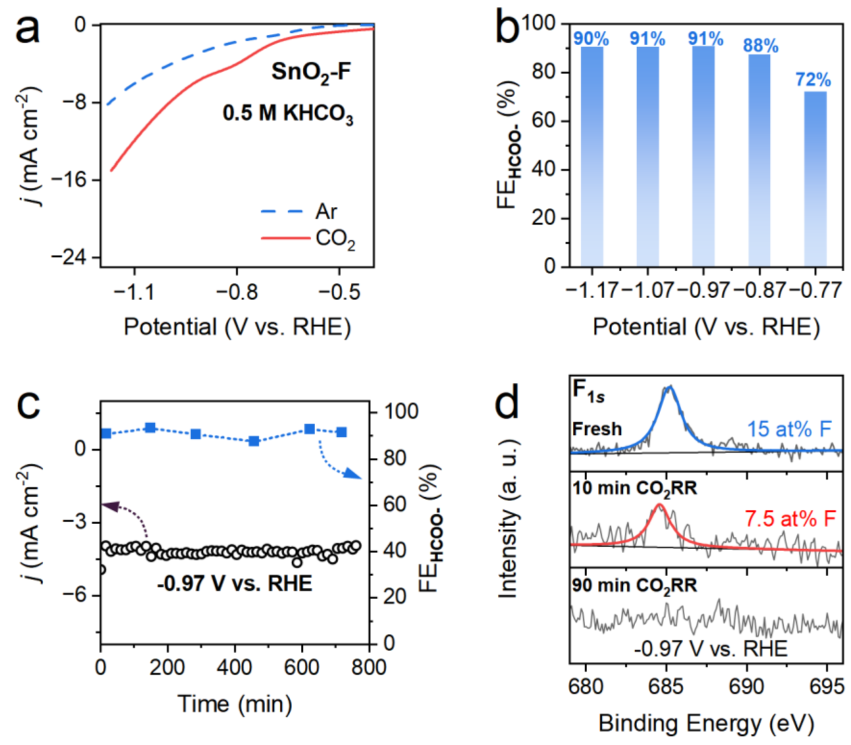

Supplementary Fig. 30. **Dopant leaching in fluorinated tin oxide (SnO_2-F).** (a) LSV of SnO_2-F with pH corrections for CO_2 and Ar saturated electrolytes. (b) FE_{HCOO-} of SnO_2-F at different applied potentials ranging from -0.77 V to -1.17 V (RHE). (c) Long-term durability of formate selectivity of the SnO_2-F under chronoamperometry test (0.5 M $KHCO_3$). (d) Time-dependent XPS of SnO_2-F after CO_2RR at -0.97 V_{RHE} (a. u. = arbitrary units).

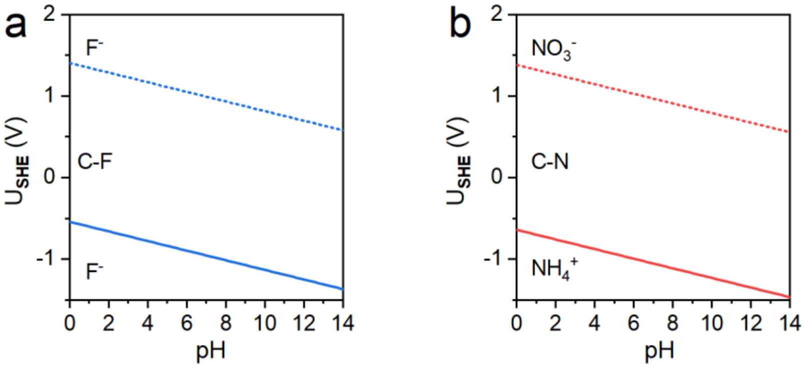

Supplementary Fig. 31. **Theoretical calculation of the stability interval of the catalyst.** Pourbaix diagram of (a) F- and (b) N-dopants.

2. Supplementary Tables

Supplementary table 1. The EXAFS spectra fitting parameters.

R: distance, CN: coordination number, E_0: energy shift, σ^2: mean-square disorder (Debye-Waller factor). Amplitude Reduction Factor S_0=0.8, Fitting R range: 1 - 4 Å, k range: 2 - 11 $Å^{-1}$.
The numbers in brackets are the last digit errors and full error (followed with *)[10].

	Scattering paths	R(Å)	CN	E_0	σ^2
Bi_2O_3	Bi-O	2.12(1)	2.7(3)	-3.58(1.39*)	0.006(1)
	Bi-Bi	3.51(2)	1	-3.58(1.39*)	0.002(1)
	Bi-Bi	3.81(4)	5.4(6)	-3.58(1.39*)	0.020(7)
Bi_2O_3-F	Bi-O	2.13(1)	2.7(4)	-2.83(1.62*)	0.006(2)
	Bi-F	2.69(0.12*)	0.2	-2.83(1.62*)	0.007(0.018*)
	Bi-Bi	3.53(4)	1	-2.83(1.62*)	0.007(4)
	Bi-Bi	3.83(4)	5.4(8)	-2.83(1.62*)	0.019(7)

Supplementary table 2. Previous CO_2RR performances (in H-cell) of the Bi-based catalysts in some related representative literature.

Catalyst	Electrolyte	Working potential (V vs RHE)	Current density (mA/cm^2)	FE_{HCOO-}	Durability	Reference
Bi_2O_3-F	0.5 M $KHCO_3$	-0.97	19.3	96.7%	100 h	This work
Bi_2O_3 NF@GDE	0.5 M $KHCO_3$	-1.0	26.1	91.7%	100 h (drop to 80% after 80 h)	ACS catal. (2021)[3]
f-Bi_2O_3@CFP	0.1 M $KHCO_3$	-1.2	24	87%	16 h	Adv. Funct. Mater. (2020)[11]
Basal-oriented BiNSs	0.5 M $KHCO_3$	-0.85	~13	93.3%		Appl. Catal. B: Environ. (2021)[12]
Bi-MOF drived 3.5 nm BiNSs	0.1 M $KHCO_3$	-1.1	~10	92%	10 h	Angew. Chem. Int. Ed. (2021)[13]
S- Bi_2O_3-CNT	0.5 M $KHCO_3$	-0.9<br>-1.2	28.2<br>48.6	97.1%<br>90%	10 h	ACS catal. (2021)[14]

Supplementary table 3. The XRD refinement results of Bi_2O_3-F, Bi-F, and $Bi_{def\text{-}F}$.

The $Bi_{def\text{-}F}$ exhibited a 1% lattice expansion along the a-axis. R_{wp}: R-weighted pattern[15].

Parameters	Bi_2O_3-F	Bi-F	$Bi_{def\text{-}F}$
a (Å)	7.73241	4.53488	4.58843
b (Å)	7.73241	4.53488	4.58843
c (Å)	5.62584	11.814	11.814
α (º)	90.0000	90.0000	90.0000
β (º)	90.0000	90.0000	90.0000
γ (º)	90.0000	120.0000	120.0000
V (Å^3)	336.370	210.406	215.405
R_{wp} (%)	3.853	3.517	2.258
x^2	2.9	6.17	2.44

Supplementary table 4. The standard electrode potentials (E^0) of half-electrochemical thermodynamic reactions.

The points near the standard electrode potentials (-0.5 V, 0 V, and 1 V vs. RHE) can be chosen as the dividing point for the interval sets of reaction testing window[16].

half-electrochemical reactions	E^0 (V vs. SHE)	E^0 (V vs. RHE)	Typical electrolyte pH
$O_2+4H^++4e^-\rightarrow 2H_2O$	1.229	1.288	1
$O_2+2H_2O+4e^-\rightarrow 4OH^-$	0.401	1.168	13
$2H^++2e^-\rightarrow H_2$	0	0.059	1
$2H_2O+2e^-\rightarrow H_2+2OH^-$	-0.828	-0.061	13
$CO_2+2HCO_3^-+2e^-\rightarrow CO+H_2O+2CO_3^{2-}$	-0.716	-0.300	7
$CO_2+H_2O+2e^-\rightarrow CO+2OH^-$	-0.934	-0.521	7
$CO_2+HCO_3^-+2e^-\rightarrow HCOO^-+CO_3^{2-}$	-1.030	-0.617	7
$CO_2+H_2O+2e^-\rightarrow HCOO^-+OH^-$	-1.078	-0.665	7

3. References

1. Ravel B, Newville M. ATHENA, ARTEMIS, HEPHAESTUS: data analysis for X-ray absorption spectroscopy using IFEFFIT. *J. Synchrotron Rad.* **12**, 537-541 (2005).
2. Zhao J, Montano PA. Effect of the electron mean free path in small particles on the extended. *Phys. Rev. B* **40**, 3401-3404 (1989).
3. Dutta A, *et al.* A Tandem (Bi_2O_3 → Bimet) Catalyst for highly efficient ec-CO_2 conversion into formate: Operando raman spectroscopic evidence for a reaction pathway change. *ACS Catal.* **11**, 4988-5003 (2021).
4. Taylor P, *et al.* Structure, spectra, and stability of solid bismuth carbonates. *Can. J. Chem.* **62**, 2863-2873 (1984).
5. Zhu J, Mu S. Defect engineering in carbon-based electrocatalysts: Insight into intrinsic carbon defects. *Adv. Funct. Mater.* **30**, 2001097 (2020).
6. Yang S, *et al.* Overlooked role of nitrogen dopant in carbon catalysts for peroxymonosulfate activation: Intrinsic defects or extrinsic defects? *Appl. Catal. B: Environ.* **295**, 120291 (2021).
7. Bondue CJ, *et al.* Suppression of hydrogen evolution in acidic electrolytes by electrochemical CO_2 reduction. *J. Am. Chem. Soc.* **143**, 279-285 (2021).
8. Liu J, *et al.* High performance platinum single atom electrocatalyst for oxygen reduction reaction. *Nat. Commun.* **8**, 15938 (2017).
9. Liu J, *et al.* High-performance oxygen reduction electrocatalysts based on cheap carbon black, nitrogen, and trace iron. *Adv. Mater.* **25**, 6879-6883 (2013).
10. Gong Q, *et al.* Structural defects on converted bismuth oxide nanotubes enable highly active electrocatalysis of carbon dioxide reduction. *Nat. Commun.* **10**, 2807 (2019).
11. Tran-Phu T, *et al.* Nanostructured β-Bi_2O_3 fractals on carbon fibers for highly selective CO_2 electroreduction to formate. *Adv. Funct. Mater.* **30**, 1906478 (2020).
12. Dan W, *et al.* Unravelling the electrocatalytic activity of bismuth nanosheets towards carbon dioxide reduction: Edge plane versus basal plane. *Appl. Catal. B: Environ.* **299**, 120693 (2021).
13. Yao D, *et al.* The controllable reconstruction of Bi-MOFs for electrochemical CO_2 reduction through electrolyte and potential mediation. *Angew. Chem. Int. Ed.* **60**, 18178-18184 (2021).
14. Liu S-Q, *et al.* Electronic delocalization of bismuth oxide induced by sulfur doping for efficient CO_2 electroreduction to formate. *ACS Catal.* **11**, 7604-7612 (2021).
15. Toby BH, Von Dreele RB. GSAS-II: the genesis of a modern open-source all purpose crystallography software package. *J. Appl. Crystallogr.* **46**, 544-549 (2013).
16. Qiao J, *et al.* A review of catalysts for the electroreduction of carbon dioxide to produce low-carbon fuels. *Chem. Soc. Rev.* **43**, 631-675 (2014).
